# Age-Specific Frequencies and Characteristics of Ovarian Cysts in Children and Adolescents

**DOI:** 10.4274/jcrpe.3781

**Published:** 2017-03-01

**Authors:** Hamdi Cihan Emeksiz, Okşan Derinöz, Esra Betül Akkoyun, Faruk Güçlü Pınarlı, Aysun Bideci

**Affiliations:** 1 Gazi University Faculty of Medicine Hospital, Department of Pediatric Endocrinology, Ankara, Turkey; 2 Gazi University Faculty of Medicine Hospital, Department of Pediatric Emergency, Ankara, Turkey; 3 Gazi University Faculty of Medicine Hospital, Department of Pediatric Oncology, Ankara, Turkey

**Keywords:** Ovarian cyst, frequency, characteristics, children, adolescents

## Abstract

**Objective:**

The aim of the present study was to document ovarian cyst frequency and characteristics as well as distribution of these parameters with respect to age in children and adolescents.

**Methods:**

We retrospectively analyzed the medical records of 1009 girls between the ages of 5-18 years who presented to our pediatric emergency department (PED) with pelvic pain and therefore underwent pelvic ultrasound examination between June 2011 and May 2014.

**Results:**

In total, 132 of 1009 girls (13.1%) were identified as having ovarian cysts ≥1 cm in diameter. The frequency of ovarian cysts was found to be 1.8% (6/337) in children aged 5-9 years and 18.8% (126/672) in those aged 10-18 years. All the cysts detected in children aged 5-9 years were small (<3 cm) and simple with age-specific frequencies ranging between 1.5-2.7%. With the onset of adolescence, ovarian cyst frequency started to increase with age and ranged between 3.8-31.3% throughout adolescence. Age of peak ovarian cyst frequency was 15 years with a rate of 31.3%. Large ovarian cysts (>5 cm) were identified in 19 adolescents (15.1%) with most occurring during middle adolescence. Of the 19 adolescents, five were found to have cyst-related significant ovarian pathologies including cystadenoma (n=3) and ovarian torsion (n=2).

**Conclusion:**

In children aged 5-9 years, ovarian cysts were infrequent and small (<3 cm). Peak ovarian cyst frequency was detected at the age of 15 years. All patients diagnosed with cyst-related significant ovarian pathologies were adolescents having a cyst >5 cm in diameter with a complex appearance in most.

**WHAT IS ALREADY KNOWN ON THIS TOPIC?**

The discovery rate of ovarian cysts in children and adolescents has remarkably increased in parallel to the increased usage of ultrasonography in pediatric imaging. The vast majority of the ovarian cysts detected in children and adolescents are asymptomatic functional (physiologic) cysts with simple appearance and small size (1-3 cm). Although ovarian cysts are far more common and larger in adolescents than in children due to the increased hormonal stimulation of ovaries during puberty, there is still limited data available about the epidemiology and characteristics of ovarian cysts in the pediatric population.

**WHAT THIS STUDY ADDS?**

The present study documented the age-specific frequencies and characteristics of ovarian cysts in children and adolescents. The findings may contribute to the understanding of normal ovarian developmental process and may help improve the management of ovarian cysts in the pediatric population.

## INTRODUCTION

Widespread use and availability of ultrasonography in pediatric imaging has led to an increase in the number of detected ovarian cysts in children suggesting that they are more common than considered in the pediatric population. The great majority of ovarian cysts are asymptomatic functional (physiologic) cysts with simple appearance and small size (1-3 cm), but sometimes they grow to larger sizes (>5 cm) and rarely become clinically evident by being ruptured or causing ovarian torsion (1,2). Although ovarian cysts are known to be more common and larger in adolescents compared to children as a result of increased gonadotropin stimulation of ovaries during puberty (3), very limited data exist about the epidemiology and characteristics of ovarian cysts in the pediatric population. There are only a few studies on the incidence and ultrasound findings of ovarian cysts in children and adolescents. Millar et al (4) found ovarian cysts in 2%-5% of females between the ages of 0-8 years, the majority of these cysts being insignificant and small, less than 1 cm in diameter. In adolescents, Porcu et al (5) reported that the frequency of ovarian cysts >3 cm was 12% in their series of 139 girls aged 10-19 years. Most of these cysts were considered to be functional cysts.

The aim of the present study was to document ovarian cyst frequency, size, and characteristics, as well as distribution of these parameters with respect to age in children and adolescents. Such information may contribute to the understanding of normal ovarian developmental process and may help improve the management of pediatric ovarian cysts.

## METHODS

We retrospectively reviewed the records of girls between the ages of 5-18 years who presented to our pediatric emergency department (PED) at Gazi University Faculty of Medicine Hospital, PED, Ankara, Turkey, between June 01, 2011 and May 31, 2014 because of pelvic pain and therefore underwent pelvic ultrasound examination. During that time interval, a total of 1157 girls (5-18 years of age) were subjected to pelvic ultrasound examination in PED. One hundred forty-eight girls who had chromosomal abnormalities, a history of chronic illness or cancer, and those whose one or both ovaries could not be seen on ultrasound were excluded from the study. Finally, 1009 girls who met the inclusion criteria were enrolled, and 132 of these 1009 girls were found to have ovarian cysts incidentally or purposely in ultrasound examination. Characteristics of the ovarian cysts including size, laterality, appearance, and frequency of ovarian cyst formation for each age and for growth stages [childhood (<10 years), early adolescence (10-14 years), middle adolescence (15-17 years), and late adolescence (18-19 years)] were analyzed in the present study. The ultrasound system used in the sonographic examination of the pelvic organs was a GE Logiq Ultrasound device (March 2009, Wisconsin, USA) with a 5-2-MHz convex transducer. All girls were scanned with a full bladder. A simple cyst was defined as having anechoic fluid, a smooth thin wall, posterior acoustic enhancement, and no solid component or septation. An ovarian cyst with a solid component and/or septation, and/or internal echoes and/or echogenic wall was considered as a complex ovarian cyst (1). The size of an ovarian cyst was defined as the maximum measurement in any dimension and was categorized into three groups: <3 cm, 3-5 cm, and >5 cm. In girls with bilateral ovarian cysts ≥1 cm, characteristics of the largest cyst were recorded. Cysts smaller than 1 cm in diameter (microcysts-ovarian follicles) were not included in the study. The study protocol was approved by Clinical Trials Ethics Committee of Gazi University Faculty of Medicine (#01262015-43).

### Statistical Analysis

The statistical analysis was performed using the Package for Social Sciences (SPSS) software (version 16, SPSS Inc. Chicago, IL, USA). A descriptive analysis was used to determine the characterization of patients and ovarian cysts. Descriptive statistics were expressed as frequency and percentage for categorical variables, whereas quantitative data were expressed as mean standard deviation for normally distributed variables and as medians (minimum-maximum) for non-normally distributed data.

## RESULTS

During a 3-year time span, of 1009 girls (672 adolescents) aged 5-18 years, 132 (13.1%) were identified as having an ovarian cyst ≥1 cm. The frequency of ovarian cysts was found to be 1.8% (6/337) in children aged 5-9 years and 18.8% (126/672) in adolescents aged 10-18 years. Age-specific frequencies of ovarian cyst formation and their distribution with respect to cyst size are summarized in Table 1. Age-specific cyst frequencies in children aged 5-9 years varied between 1.5-2.7%. All the ovarian cysts detected in these children were small (<3 cm). With the onset of adolescence, ovarian cyst frequency started to rise and to reach ranges of 3.8-30.9% in early adolescence, 26.7-31.3% in middle adolescence, and 13.6% in late adolescence. Peak ovarian cyst frequency was at age 15 years with a rate of 31.3%. Of the 126 adolescents with an ovarian cyst, 19 (15.1%) were found to have large ovarian cysts (>5 cm) occurring mostly during middle adolescence.

Sonographic characteristics of ovarian cysts by age groups are given in Table 2. Most of the cysts were right-sided (59.8%), simple (68.9%), and <3 cm in diameter (58.3%). Of the 19 large ovarian cysts, 11 (57.9%) were right-sided and 14 (73.7%) were complex. Seven adolescents were found to have a complicated ovarian cyst presenting with ovarian torsion (n=2) and ruptured cyst (n=5). The frequency of ovarian torsion was 10.5% (2/19) in adolescent girls with a cyst >5 cm in diameter. Of the two girls with ovarian torsion, one had a simple right-sided ovarian cyst with a size of 7 cm and presented with right lower quadrant pain and vomiting, while the other girl had a complex left-sided cyst with a size of 5.1 cm and presented with left lower quadrant pain. Both underwent cystectomy and detorsion. Girls with ruptured cysts were all hemodynamically stable.

Three adolescents had benign neoplastic ovarian cysts measuring >5 cm in diameter, identified as mucinous cystadenoma (n=2) and serous cystadenoma (n=1). All underwent salpingooophorectomy. Their tumor markers including alpha-fetoprotein (AFP), beta-human chorionic gonadotropin (β-HCG), cancer antigen 125 (CA-125), lactate dehydrogenase (LDH), and carcinoembryonic antigen (CEA) were all in normal ranges (Table 3). The patient with mucinous cystadenoma measuring 21 cm in diameter had an additional symptom of abdominal fullness. The two patients with mucinous cystadenoma underwent surgery soon after the sonographic examination due to the enormous size (≥15 cm) and appearance of the cysts, and the patient with serous cystadenoma, after 6 months of follow-up due to the persistence of the cyst.

## DISCUSSION

In our study, 132 girls (13.1%) aged between 5 and 18 years were found to have an ovarian cyst ≥1 cm in diameter. Age-specific frequencies of ovarian cysts were low and almost constant during childhood. With the onset of early adolescence, the frequency of ovarian cyst formation started to rise and made a peak by the age of 15 years and remained roughly elevated for all cyst size categories throughout the middle adolescence. All girls who were found to have a cyst-associated significant ovarian pathology including ovarian torsion or neoplasm were adolescents having a large ovarian cyst with a complex appearance in most.

In the present study, the frequency of ovarian cysts ≥1 cm was found to be 1.8% in children aged 5-9 years and age-specific cyst frequencies in this age interval varied between 1.5-2.7%. Thus far, the few studies documenting the frequency of ovarian cyst formation in children reported largely varying rates most probably due to the differences between the studies in terms of study design and sample size. Millar et al (4) retrospectively analyzed 1818 pelvic ultrasonography findings in prepubertal girls aged 0-8 years and found that the frequency of ovarian cysts<1 cm in diameter was 2-5%, varying with respect to age group. Ovarian cysts >2 cm were very rare in their series and detected in 0.9% of prepubertal girls and in only 0.2% of girls who were older than 2 years of age. However, the incidence of ovarian cysts between 1 cm and 2 cm in diameter was not reported in this study. We found age-specific frequencies of ovarian cysts of ≥1 cm in diameter almost constant and low from 5 years to 9 years of age reflecting the relatively dormant status of ovaries during childhood. All the cysts detected in children aged 5-9 years were <3 cm in diameter, simple and uncomplicated.

The ovary is more active during puberty due to the increased gonadotropin secretion. Hence, finding an ovarian cyst is more common in adolescence than in any other stage of growth. However, very limited data are available on the incidence and characteristics of ovarian cysts in adolescents. Porcu et al (5) followed 139 adolescent girls aged between 10 and 19 years with serial ultrasound assessment in the follicular phase of the menstrual cycle and reported that the incidence of ovarian cyst formation >3 cm was 12%. This was higher than the rate (8.2%) we found in adolescents for ovarian cysts >3 cm. The difference in frequencies between the two studies may be related to their design, sample size, and ethnicity dissimilarities, as well as to the unmentioned age-specific distribution of adolescent girls in the study of Porcu et al (5), since clustering of their cases particularly at perimenarcheal ages would likely lead to the overestimation of the ovarian cyst incidence in adolescent girls. Kanizsai et al (6) studied the characteristics of ovarian cysts in 119 girls undergoing ultrasound examination due to irregular bleeding in most and reported that the majority of the cysts they detected in their obstetrics and gynecology clinic were simple and unilateral, findings which are similar to those in our study. They also categorized the cysts with respect to their size and found that most of the cysts (63%) were between 3-5 cm in diameter followed by 28% >5 cm, and 9% between 1-3 cm. In contrast to these findings, more than half of the cysts (58%) in our series were found to be <3 cm in diameter, 27% between 3-5 cm, and 15% >5 cm. The frequency of ovarian cysts measured between 3-5 cm seems to be higher than normal in the series of Kanizsai et al (6) since most ovarian cysts detected in premenopausal women are functional cysts (follicular or corpus luteum) which are typically not larger than 3 cm in diameter (7). In addition, girls in the Kanizsai et al (6) study were evaluated for more typical complaints associated with ovarian disorders, as well as in a more competent division, both of which consequentially may have led to the overestimation of the frequency of ovarian cysts >3 cm in their study.

The number and size of ovarian follicles vary depending on the stage of puberty (3). In the years just before menarche, some follicles exceed 1 cm in diameter and become ovarian cysts with a more prevalent fluid component. These cysts can reach larger sizes which then often spontaneously regress (6,8). Accordingly, in the present study, we found that the frequency of ovarian cyst formation began to rise by the age of 11 years which is nearly 1.7 years before the average age of menarche in Turkish girls (9). At perimenarcheal ages (within 3 years of menarche) the formation of ovarian cysts becomes more prominent because of considerably high number of antral follicles in this period of life and their increased sensitivity to mature gonadotropin stimulation as well (10,11,12). Moreover, the early period following menarche is commonly associated with anovulatory cycles. Therefore, ovulatory cysts are often seen as the result of aborted ovulations or corpus luteum persistence and this is the reason why both follicular and corpus luteal cysts are extremely common in this period of life (2). Consistent with these data, we found that the frequency of ovarian cyst formation made a remarkable peak by the age of 15 years (31.3%) and remained almost elevated for all cyst size categories throughout middle adolescence. However, with the onset of late adolescence, ovarian cyst frequency was found to follow a downward trend. The latter finding may be due to the completion of the ovarian maturation process in almost all girls by the end of middle adolescence.

Cysts greater than 5 cm pose a higher risk for torsion (2). Likewise, two adolescent girls who were identified to have ovarian torsion in our series had a cyst diameter >5 cm. The torsion rate was found to be 10.5% (2/19) in girls with an ovarian cyst >5 cm. Both girls were treated by cystectomy and detorsion. Five adolescents were sonographically detected to have ruptured ovarian cysts. All were hemodynamically stable.

After mature teratomas, cystadenomas are the second most common benign ovarian tumors in children and adolescents (13). They can be either serous or mucinous and can grow up to enormous sizes. Mucinous cystadenomas tend to be much larger than serous cystadenomas at presentation (14). Three girls with a cyst size >5 cm were found to have unilateral cystadenomas including 2 mucinous and 1 serous type. Consistent with the literature, both mucinous cystadenomas were larger than the serous one. Girls with mucinous cystadenoma underwent surgery soon after the sonographic examination due to the enormous size (≥15 cm) and appearance of the cysts. Surgery was performed in the girl with serous cystadenoma after 6-month follow-up due to the persistence of the cyst. Tumor markers studied were not helpful in the diagnosis of these tumors.

Our study has several limitations. It was retrospective in design, data about pubertal stage of the girls at presentation and menstruation history of the adolescents having periods were unavailable. Also, the ultrasonographic examinations were performed by different radiologists. Despite these handicaps, to our knowledge, this is the first study to report age-specific frequencies and characteristics of ovarian cysts in adolescence, and our findings may help to improve the management of future cases of adolescents with cyst-related disorders.

In conclusion, this study presented descriptive data on pediatric ovarian cyst frequency, size, and characteristics, as well as distribution of these parameters with respect to age in a relatively large cohort of girls from Turkey. In children aged 5-9 years; ovarian cysts were infrequent and small (<3 cm), suggesting that ovarian cyst-related urgent conditions seem to be rare in this period of life. With the onset of adolescence, ovarian cyst frequency increased with age and made a peak by the age of 15 years and remained roughly elevated for all cyst size categories throughout middle adolescence, presumably as a consequence of normal ovarian developmental process. All patients diagnosed with a cyst-related significant ovarian pathology were adolescents having a cyst >5 cm in diameter with a complex appearance in most.

## Figures and Tables

**Table 1:**
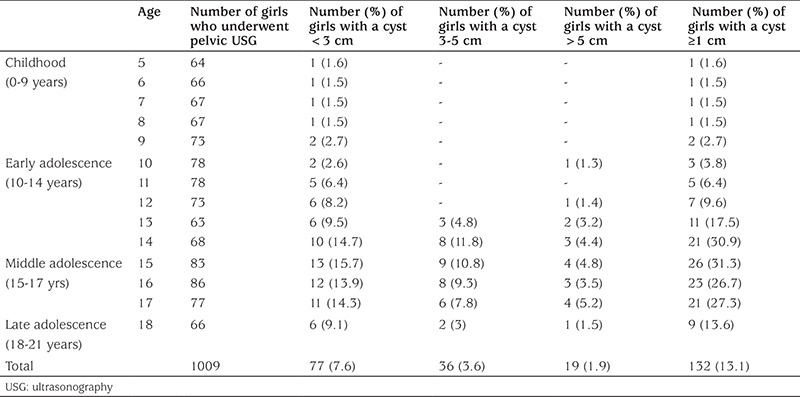
Ovarian cysts in children and adolescents

**Table 2:**
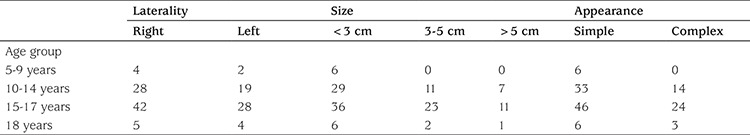
Characteristics of ovarian cysts by age groups

**Table 3:**
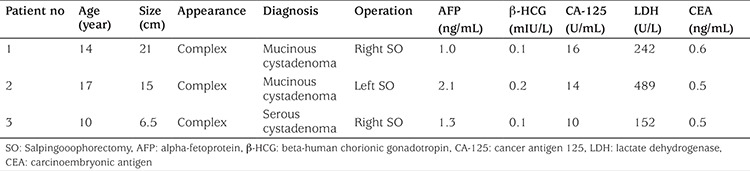
Summary of patients with benign ovarian neoplasms
